# Optimal Bowel Preparation for Video Capsule Endoscopy

**DOI:** 10.1155/2016/6802810

**Published:** 2015-12-31

**Authors:** Hyun Joo Song, Jeong Seop Moon, Ki-Nam Shim

**Affiliations:** ^1^Department of Internal Medicine, Jeju National University School of Medicine, Jeju, Republic of Korea; ^2^Department of Internal Medicine, Inje University College of Medicine, Seoul, Republic of Korea; ^3^Department of Internal Medicine, Ewha Womans University School of Medicine, Seoul, Republic of Korea

## Abstract

During video capsule endoscopy (VCE), several factors, such as air bubbles, food material in the small bowel, and delayed gastric and small bowel transit time, influence diagnostic yield, small bowel visualization quality, and cecal completion rate. Therefore, bowel preparation before VCE is as essential as bowel preparation before colonoscopy. To date, there have been many comparative studies, consensus, and guidelines regarding different kinds of bowel cleansing agents in bowel preparation for small bowel VCE. Presently, polyethylene glycol- (PEG-) based regimens are given primary recommendation. Sodium picosulphate-based regimens are secondarily recommended, as their cleansing efficacy is less than that of PEG-based regimens. Sodium phosphate as well as complementary simethicone and prokinetics use are considered. In this paper, we reviewed previous studies regarding bowel preparation for small bowel VCE and suggested optimal bowel preparation of VCE.

## 1. Introduction

Video capsule endoscopy (VCE) is useful in investigating small bowel as well as esophagus, stomach, and colon. Bowel preparation for small bowel VCE is recommended to improve small bowel visualization quality (SBVQ), diagnostic yield (DY), and cecal completion rate (CR). Particularly in the distal small bowel, DY of VCE can be limited due to reduced SBVQ-associated with residual material or dark colored bile. According to a 2009 meta-analysis of 12 studies [1], purgative bowel cleansing prior to VCE improves the SBVQ and increases the DY but does not alter the VCE CR. However, the gastric transit time (GTT) and small bowel transit time (SBTT) of VCE were not affected by purgatives.

We performed online search for VCE bowel preparation-related clinical studies, comparative research, randomized controlled trials (RCTs), meta-analyses, and guidelines published from January 2002 to June 2015. Literature review was conducted using Key MeSH terms of “capsule endoscopy” and “bowel preparation.” We also reviewed bowel preparation guidelines for VCE of small bowel based on 2009 European Society of Gastrointestinal Endoscopy (ESGE) guidelines [2], 2013 ESGE guidelines [3], and 2013 Korean guidelines [4] by the Korean Gut Image Study Group, part of the Korean Society of Gastrointestinal Endoscopy. The level of scientific evidence for recommendation was based on study design; for example, the evidence of randomized trial was considered high, observation study was low, and any other type of evidence was very low. The validity of the recommendation was divided into categories of “strong” or “weak” (Table 1) [5]. In this paper, we introduced previous studies on bowel preparation for VCE and suggested optimal preparation methods.

## 2. Purgatives

### 2.1. Polyethylene Glycol

Polyethylene glycol- (PEG-) based regimens are first-line recommendation (Grade A) [3]. The majority of the evidence of bowel preparation prior to small bowel VCE is PEG-based regimens. The 2009 ESGE guidelines recommended purgative bowel preparations in order to enhance small bowel DY by VCE without affecting the CR (category of evidence, 2a; grade of recommendation, B) [2]. According to the Korean Gut Image Study Group guidelines [4], bowel preparation with PEG solution enhances DY and SBVQ, without effect on cecal CR (strong recommendation, moderate quality evidence).

Table 2 shows many studies regarding bowel preparation with comparison of PEG versus clear liquid or fasting for small bowel VCE, including prospective randomized controlled trials [6–13], a prospective blinded nonrandomized trial [14], and a retrospective study [15]. Most studies were performed by comparing SBVQ, DY, and cecal CR between 2 L PEG solution and clear diet or fasting groups. Four-liter PEG solution was used in a few studies [10, 14]. In addition, ingestion of a small amount of PEG (500 mL) beginning 30 minutes after swallowing the capsule significantly improves SBVQ and cecal CR, although DY was not affected [8]. Another study regarding a small amount (500 mL) of PEG solution over 2 hours, beginning 30 minutes after swallowing the capsule, showed increased SBVQ without any difference in cecal CR [12]. Since PEG is completely transparent, a view through PEG was considered better than a view through natural intestinal fluid. However, negative result regarding SBVQ with 2 L PEG was reported in one retrospective study [15].

Two-liter PEG solution bowel preparation is similar to that of 4 liters of PEG in DY, SBVQ, and CR of VCE (weak recommendation, moderate quality evidence). Two studies by Kantianis et al. [16] and Park et al. [11] indicated no significant difference between 2 L and 4 L PEG in regard to small bowel cleansing and CR. Therefore, 2 L PEG should be recommended as preparation for VCE, administered on the day prior to the procedure, as the most commonly used preparation method [17].

In colonoscopy, bowel preparation status is classified as excellent, good, fair, poor, or inadequate. Clinically, most gastroenterologists considered excellent and good bowel preparation status as optimal bowel preparation. However, there was no consensus of optimal bowel preparation for VCE, as each study with PEG suggested various definitions for bowel preparation quality (Table 3). A recent study considered excellent or good preparation (>75% small bowel visualization) as adequate bowel preparation [13]. Therefore, standardized definition of optimal bowel preparation for VCE is necessary.

To date, there has been no consensus regarding optimal timing of bowel preparation for VCE. To evaluate optimal timing of VCE bowel preparation, a single-center randomized controlled trial was conducted by Black et al. [18]. However, there was no significant difference between the quality and timing (day before VCE versus 4 hours prior to VCE) of small bowel preparation. Intestinal lavage administered one day prior was similar to same-day preparation with regard to overall preparation quality, SBTT, frequency of identified mucosal abnormalities, general DY, and CR. One of the issues for bowel preparation of VCE is that the distal segment of the small intestine should be improved. The main limitation of this study is that the number of patients (*n* = 34) is not sufficient for generalizing to actual practice. Therefore, multicenter large randomized controlled trial is required to clarify optimal timing of bowel preparation for VCE.

According to the 2012 consensus guidelines for bowel preparation [19], purgative is absolutely contraindicated in patients with gastrointestinal obstruction, ileus, ulcer, perforation, or inflammatory bowel diseases. In addition, it is also contraindicated in patients with decreased consciousness, swallowing disorders, and hypersensitivity to oral bowel-cleansing agents and in patients having an ileostomy. Therefore, optimal bowel preparation should be made considering individual patient risk factors.

### 2.2. Sodium Picosulfate

Recently, various types of bowel preparation such as PEG, PEG plus ascorbic acid, sodium picosulfate, and phosphate (NaP) are available. There has been no published evidence to support the use of sodium picosulfate; however, it is used in some units. Anecdotal evidence suggests that it is not as effective as PEG [3].

### 2.3. Sodium Phosphate (NaP)

NaP is not recommended for bowel cleansing due to the potential for renal damage and other adverse effects (Grade B) [3]. However, the use of NaP is possible in patients for whom PEG or sodium picosulfate is ineffective or not tolerated (Grade D). According to a previous study conducted using NaP, SBVQ of NaP group is better than overnight fasting (35% versus 4%) [20]. However, recent meta-analysis of NaP-based regimens revealed no significant difference from fasting alone (OR = 1.32, 95% CI = 0.52–2.96, *p* < 0.0001) [21]. Therefore, NaP should not be used in general.

## 3. Simethicone

Preparing the small bowel with simethicone has been reported to increase SBVQ by reducing intraluminal air bubbles [22, 23].  Table 4 demonstrates several studies regarding bowel preparation with simethicone for small bowel VCE [13, 22–26]. Systemic review and meta-analysis of RCTs of simethicone revealed that supplemental use of simethicone prior to VCE enhances SBVQ, especially for patients without purgative, but does not affect the cecal CR [27]. It decreases air bubbles in the colonic lumen but does not improve bowel preparation. Additionally, its effect on DY remains controversial. Bowel preparation by fasting or administration of PEG solution combined with simethicone enhances SBVQ, but it does not affect CR for VCE (strong recommendation, moderate quality evidence) [4].

## 4. Prokinetics

Prokinetics can be used for shortening of the GTT and may improve cecal CR. To date, various prokinetics including erythromycin [28–30], mosapride [31], metoclopramide [32–34], and lubiprostone [35, 36] have been investigated for bowel preparation of VCE.  Table 5 exhibits previous studies regarding bowel preparation with various prokinetics for small bowel VCE. Previously, the battery time of VCE was 8 hours and approximate 20% do not reach the colon due to limited recording time [34]. Currently, the battery time of VCE is about 12 hours; therefore the effect of prokinetics on the CR could be minimal.

Lubiprostone, a selective activator of type 2 chloride channels in the apical membrane of the GI epithelium, as a propulsive agent was investigated for decreasing the SBTT by VCE. However, there were opposite results regarding the GTT and SBTT in two studies [35, 36]. Lubiprostone neither decreased the GTT and SBTT nor improved SBVQ for VCE in one double-blind placebo-controlled study [35], while it decreased the SBTT by VCE in another exploratory randomized, double-blind, controlled study [36]. Bowel preparation with prokinetics does not enhance the SBVQ, DY, or CR of VCE. Therefore, it is not generally recommended (weak recommendation, moderate quality evidence) [4].

## 5. Miscellaneous

Recently, there have been new studies using substances such as coffee enema or magnesium citrate. Coffee enema is known to induce dilation of bile ducts and excretion of bile through the colon wall. During VCE, excreted bile is one of the causes of poor bowel preparation. Coffee enema for preparation for small bowel VCE was investigated by a pilot study (*n* = 34) [37]. Comparison of coffee enema plus 2 L PEG versus 2 L PEG demonstrated greater efficacy of bowel preparations in the mid-to-distal segments of the small bowel in patients who received coffee enema plus 2 L PEG than in those who received PEG only. In one magnesium citrate trial of bowel preparation for VCE [38], there was no significant difference between the group that received the preparation (34 g magnesium) and the control group.

## 6. Conclusion

Bowel preparation is generally recommended for small bowel VCE. Currently, a combination of 2 L PEG and simethicone appears to be the optimal bowel preparation before VCE. After reviewing current articles regarding bowel preparation for VCE, we suggest using purgatives such as PEG as first line and sodium picosulfate as second line with antifoaming agent. However, sodium phosphate should not be used except for the patients whom PEG or sodium picosulfate is not effective and intolerable. However, prokinetics (erythromycin, metoclopramide, or lubiprostone) are not generally recommended. For each of these agents, including purgative (PEG, sodium picosulfate, and sodium phosphate), consensus is needed regarding optimal timing of bowel preparation. Therefore, best bowel preparation is determined considering individual patient status.

## Figures and Tables

**Table 1:** Quality of evidence and strength of a recommendation.

Quality of evidence	
High quality	Further research is very unlikely to change our confidence in the estimate of effect.
Moderate quality	Further research is likely to have an important impact on our confidence in the estimate of effect and may change the estimate.
Low quality	Further research is very likely to have an important impact on our confidence in the estimate of effect and is likely to change the estimate.
Very low quality	Any estimate of effect is very uncertain.

Strength of a recommendation	

Strong	Most or all individuals will be best served by the recommended course of action.
Weak	Not all individuals will be best served by the recommended course of action. There is a need to consider more carefully than usual individual patient's circumstances, preferences, and values.

**Table 2:** Studies comparing SBVQ, DY, and CR between PEG solution versus clear liquid or fasting of small bowel VCE.

Author (year, area)	Design	Number	PEG versus clear liquid diet or fasting		
			SBVQ	DY	CR
Viazis et al. [6] (2004, Greece)	Prospective RCT	80	90% versus 60% *p* = 0.004	65% versus 30% *p* = 0.003	80% versus 65% *p* = 0.21
van Tuyl et al. [7] (2007, Netherlands)	Prospective RCT	60	72% versus 25% *p* = 0.001	30% versus 27% *p* = 0.86	N/A
Endo et al. [8]^*∗*^ (2008, Japan)	Prospective RCT	59	N/A *p* < 0.01	78.6% versus 71.6% *p* = NS	88.9% versus 65.6% *p* = 0.038
Wi et al. [9] (2009, Korea)	Prospective RCT	99	56% versus 43% *p* = NS	50% versus 39% *p* = 0.111	71% versus 75% *p* = 0.924
Rey et al. [10] (2009, France)	Prospective RCT	116	83.1% versus 38.6% *p* < 0.05	N/A	N/A
Park et al. [11] (2011, Korea)	Prospective RCT	43	*2.43* versus 2.26 *p* = 0.045	65% versus 56.6% *p* = NS	75% versus 73% *p* = 0.869
Ito et al. [12]^*∗*^ (2012, Japan)	Prospective RCT	42	4.4 ± 0.8 versus 2.7 ± 1.0 *p* = 0.00004	N/A	85.0% versus 81.8% *p* = 0.89
Rosa et al. [13] (2013, Portugal)	Prospective RCT	60	83.3% versus 65% *p* = 0.0417	60% versus 44.4% *p* = 0.587	100% versus 88.9% *p* = 0.312
Dai et al. [14] (2005, Switzerland)	Prospective blinded nonrandomized trial	61	3.04 versus 2.41 *p* < 0.01	N/A	97% versus 76% *p* < 0.01
Ben-Soussan et al. [15] (2005, France)	Retrospective study	42	57.6% versus 62.5% *p* = NS	46.2% versus 50.0% *p* = NS	92.3% versus 100.0% *p* = NS

PEG: polyethylene glycol, VCE: video capsule endoscopy, RCT: randomized-controlled trial, SBVQ: small bowel visualization quality, DY: diagnostic yield, CR: completion rate, N/A: not applicable, and NS: no significant. ^*∗*^PEG 500 mL.

**Table 3:** Definitions of optimal bowel preparation of VCE among studies with PEG.

Author (year, area)	Design	Number	Quality of bowel preparation
Viazis et al. [6] (2004, Greece)	Prospective RCT	80	Clean: if <25% of the mucosal surface was covered by food debris or intestinal contents Adequate: if the objective score <10%
van Tuyl et al. [7] (2007, Netherlands)	Prospective RCT	60	Good visibility: visualization ≥75% of the mucosa Poor visibility: visualization <75%
Endo et al. [8]^*∗*^ (2008, Japan)	Prospective RCT	59	Percentage of visualized bowel surface area 1, <25%; 2, 25–49%; 3, 50–74%; 4, 75–89%; and 5, >90%
Wi et al. [9] (2009, Korea)	Prospective RCT	99	Clean: if <25% of the mucosal surface was covered by food debris or intestinal contents, concentrated bile, and intraluminal gas Adequate: if the objective score <10%
Rey et al. [10] (2009, France)	Prospective RCT	116	Excellent (score 4): imaging of excellent quality, all small lesions, and minor changes of the mucosa could be detected Diagnostic (score 3): imaging of sufficient quality to make an accurate diagnosis Acceptable (score 2): the imaging quality allows detection of only gross disease, and some small lesions could be missed Nondiagnostic (score 1): quality of imaging is poor; it is difficult to make a reliable final diagnosis
Park et al. [11] (2011, Korea)	Prospective RCT	43	Proportion of visualized mucosa Score 3, ≥75%; score 2, 50–75%; score 1, 25–50%; score 0, <25% Extent of obscuration by bubbles, debris and bile, and so forth. Score 3, <5%; score 2, 5–25%; score 1, 25–50%; score 0, ≥50%
Ito et al. [12]^*∗*^ (2012, Japan)	Prospective RCT	42	Percentage of visualized bowel surface area 1, <25%; 2, 25–49%; 3, 50–74%; 4, 75–89%; and 5, >90%
Rosa et al. [13] (2013, Portugal)	Prospective RCT	60	Excellent: if an ideal visualization of the small bowel mucosa was achieved Good: if >75% of the mucosa was in perfect condition Fair: if only 50%–75% of the mucosa was under perfect conditions Poor: if <50% of the mucosa could be observed Adequate: excellent or good preparation
Dai et al. [14] (2005, Switzerland)	Prospective blinded nonrandomized trial	61	Percentage of visualized bowel wall 1, <25%; 2, 25–49%; 3, 50–75%; 4, >75%
Ben-Soussan et al. [15] (2005, France)	Retrospective study	42	The presence of biliary secretion, air bubbles, and residue 1, poor; 2, fair, 3, good; 4, excellent

PEG: polyethylene glycol, VCE: video capsule endoscopy, and RCT: randomized-controlled trial. ^*∗*^PEG 500 mL.

**Table 4:** Studies comparing SBVQ, DY, and CR between simethicone versus clear liquid or fasting of small bowel VCE.

Author (year, area)	Design	Number	Simethicone versus clear liquid diet or fasting		
			SBVQ	DY	CR
Albert et al. [22] (2004, Germany)	Prospective RCT	36	72% versus 22% *p* = 0.001	N/A	N/A
Ge et al. [23] (2006, China)	Prospective RCT	56	57% versus 25% *p* = 0.0175	N/A	64.3% versus 75% *p* = NS

Author (year, area)	Design	No.	PEG + simethicone versus clear liquid diet or fasting		
			SBVQ	DY	CR

Fang et al. [25] (2009, China)	Prospective RCT	64	98% versus 68% *p* = 0.001	N/A	N/A
Spada et al. [26] (2010, Italy)	Prospective RCT	58	42% versus 43% *p* = 0.86	62% versus 72.4% *p* = 0.39	66.6% versus 70% *p* = 0.78
Rosa et al. [13] (2013, Portugal)	Prospective RCT	60	68.4% versus 65% *p* = 0.0417	57.8% versus 44.4% *p* = 0.587	89.5% versus 88.9% *p* = 0.312

PEG: polyethylene glycol, VCE: video capsule endoscopy, RCT: randomized-controlled trial, SBVQ: small bowel visualization quality, DY: diagnostic yield, CR: completion rate, N/A: not applicable, and NS: no significant.

**Table 5:** Studies comparing SBVQ, DY, and CR between prokinetics versus clear liquid or fasting of small bowel VCE.

Author (year, area)	Design	Number	Prokinetics	Prokinetics versus placebo or fasting				
				GTT (min)	SBTT (min)	SBVQ	DY	CR
Leung et al. [28] (2005, China)	Prospective nonrandomized study	38	Erythromycin	16 versus 70 *p* = 0.005	227 versus 183 *p* = 0.18	54% versus 64% *p* = 0.74	N/A	96% versus 79% *p* = 0.13
Caddy et al. [29] (2006, Australia)	Prospective RCT	45	Erythromycin	51 versus 38 *p* = 0.42	304 versus 302 *p* = 0.96	1.9 versus 2.2 *p* = 0.24	N/A	68% versus 78% *p* = 0.45
Niv et al. [30] (2008, Israel)	Retrospective blind study	100	Erythromycin	21 versus 28 *p* = 0.07	279 versus 270 *p* = 0.83	2.8 versus 2.8 *p* = 0.73	48% versus 36% *p* = N/A	90% versus 84% *p* = 0.37
Wei et al. [31] (2007, China)	Prospective RCT	60	Mosapride	14 versus 34 *p* = 0.035	248 versus 281 *p* = 0.3492	N/A	73% versus 50% *p* = 0.110	93% versus 67% *p* = 0.021
Selby [32] (2005, Australia)	Prospective RCT	150	Metoclopramide	31 versus 48 *p* = 0.025	231 versus 256 *p* = 0.35	100% versus 69% *p* = 0.998	51% versus 57% *p* = N/A	97% versus 76% *P* < 0.001
Postgate et al. [33] (2009, UK)	Prospective RCT	74	Metoclopramide	17 versus 17 *p* = 0.62	260 versus 278 *p* = 0.91	38 versus 37 *p* = 0.18	26% versus 35% *p* = 0.45	85% versus 89% *p* = 0.74
Almeida et al. [34] (2010, Portugal)	Prospective RCT	95	Metoclopramide	26 versus 28 *p* = 0.511	221 versus 256 *p* = 0.083	55% versus 54% *p* = 0.545	68% versus 65% *p* = 0.443	81% versus 77% *p* = 0.422
Hooks III et al. [35] (2009, Netherlands)	Prospective RCT	40	Lubiprostone	126 versus 43 *p* = 0.0095	188 versus 219 *p* = 0.130	NS	N/A	N/A
Matsuura et al. [36] (2014, Japan)	Prospective RCT	6	Lubiprostone	58 versus 23 *p* = 0.846	111 versus 179 *p* = 0.042	3.76 versus 2.88 *p* < 0.001	N/A	N/A

VCE: video capsule endoscopy, GTT: gastric transit time, SBTT: small bowel transit time, SBVQ: small bowel visualization quality, DY: diagnostic yield, CR: completion rate, RCT: randomized controlled trial, N/A: not applicable, and NS: no significant.
